# 一种新型中药配方通过触发磷酸戊糖途径依赖的氧化应激抑制非小细胞肺癌

**DOI:** 10.3779/j.issn.1009-3419.2023.101.27

**Published:** 2023-09-20

**Authors:** Chuan CHENG, Wei WU, Jiaxin YU, Dongdong YUAN, Yujiong WANG, Le LI

**Affiliations:** ^1^750021 银川，宁夏大学生命科学学院; ^1^School of Life Sciences, Ningxia University; ^2^西部特色生物资源保护与利用教育部重点实验室; ^2^Key Lab of Ministry of Education for Protection and Utilization of Special Biological Resources in Western China, Yinchuan 750021, China

**Keywords:** 肺肿瘤, 中药配方, 网络药理学, 氧化应激, 磷酸戊糖途径, Lung neoplasms, Chinese medicine formula, Network pharmacology, Oxidative stress, Pentose phosphate pathway

## Abstract

**背景与目的** 非小细胞肺癌（non-small cell lung cancer, NSCLC）是世界范围内死亡率较高的恶性肿瘤之一。一种新型中药配方（novel Chinese medicine formula-01, NCHF-01）在治疗NSCLC方面已显示出显著的临床疗效，但此配方治疗NSCLC的作用机制尚不完全清楚。本研究拟探讨中药配方抑制NSCLC的作用及其分子机制。**方法** 建立Lewis肺癌细胞（lewis lung cells, LLC）荷瘤小鼠，检测NCHF-01抑制肿瘤的效果；苏木素-伊红（hematoxylin-eosin staining, HE）染色检测LLC荷瘤小鼠组织器官的形态变化；NCHF-01处理NSCLC细胞，MTT和结晶紫染色实验检测其对细胞活力和增殖的影响；流式细胞术检测细胞周期、凋亡和活性氧（reactive oxygen species, ROS）水平；网络药理学预测其抑制NSCLC的作用机制；Western blot和免疫组织化学（immunohistochemistry, IHC）检测相关蛋白的表达。**结果** NCHF-01能够抑制LLC荷瘤小鼠肿瘤的生长，并对其他组织器官无明显毒副作用；NCHF-01能够抑制细胞活力和增殖，诱导细胞的G_2_/M期停滞和细胞凋亡，促进细胞内ROS水平的升高；网络药理学分析显示，NCHF-01通过氧化应激和中枢碳代谢等多种生物学过程发挥抗NSCLC作用；NCHF-01能够降低磷酸戊糖途径（pentose phosphate pathway, PPP）关键酶6-磷酸葡萄糖脱氢酶（6-phosphate glucose dehydrogenase, G6PD）和6-磷酸葡萄糖酸脱氢酶（6-phosphogluconate dehydrogenase, 6PGD）的蛋白表达以及酶活性。**结论** NCHF-01能够通过PPP依赖的氧化应激抑制NSCLC。

## Abstract (en)

**Background and objective** Non-small cell lung cancer (NSCLC) is one of the most lethal malignancies worldwide. A novel Chinese medicine formula-01 (NCHF-01) has shown significant clinical efficacy in the treatment of NSCLC, but the mechanism of this formula in the treatment of NSCLC is not fully understood. The aim of this study is to investigate the molecular mechanism of NCHF-01 in inhibiting NSCLC. **Methods ** Lewis lung cells (LLC) tumor bearing mice were established to detect the tumor inhibitory effect of NCHF-01. The morphological changes of tissues and organs in LLC tumor-bearing mice were detected by hematoxylin-eosin (HE) staining. NSCLC cells were treated by NCHF-01. The effects of cell viability and proliferation were detected by MTT and crystal violet staining experiment. Flow cytometry was used to detect cell cycle, apoptosis and reactive oxygen species (ROS). Network pharmacology was used to predict the mechanism of its inhibitory effect of NSCLC. Western blot and immunohistochemistry (IHC) were used to detect the expression of related proteins. **Results** NCHF-01 can inhibit tumor growth in LLC tumor-bearing mice, and has no obvious side effects on other tissues and organs. NCHF-01 could inhibit cell viability and proliferation, induce G_2_/M phase arrest and apoptosis, and promote the increase of ROS level. Network pharmacological analysis showed that NCHF-01 exerts anti-NSCLC effects through various biological processes such as oxidative stress and central carbon metabolism. NCHF-01 can reduce the protein expression and enzyme activity of the key enzymes 6-phosphate glucose dehydrogenase (G6PD) and 6-phosphogluconate dehydrogenase (6PGD) in the pentose phosphate pathway (PPP). **Conclusion ** NCHF-01 can inhibit NSCLC through oxidative stress dependent on the PPP.

肺癌是最常见的癌症之一，占总病例的11.4%，是全球癌症相关死亡的主要原因^[1,2]^。非小细胞肺癌（non-small cell lung cancer, NSCLC）占所有肺癌病例的80%-85%^[3]^，具有易转移、预后差、基因突变概率大等特点。尽管在开发新的化疗靶向药物和免疫检查点抑制剂方面取得了进展，但为改善治疗结果，仍有许多工作要做^[4]^。为了提高肺癌患者的治愈率，探索新的抗肺癌策略势在必行^[5]^。

在治疗癌症方面，中草药因其在预防肿瘤发生、降低毒性、提高放化疗疗效、减少肿瘤复发和转移等方面的优势，越来越受到世界各国的认可^[6]^。中草药为癌症治疗提供了大量的自然资源。然而，应用严格和系统的方法来评估中药配方的疗效并将其转化为临床药物至关重要^[7⇓⇓-10]^。本研究所使用的中药配方（novel Chinese medicine formula-01, NCHF-01）由苦参、虎杖、紫草、连翘、桂枝等12味中药组成，该方有通肺清热化痰之功效。虽然NCHF-01已被用于肺癌的临床治疗，但其抗NSCLC的机制尚不清楚。因此，本研究使用NCHF-01通过体内外实验，基于网络药理学探讨NCHF-01抗NSCLC的作用机制，为NCHF-01临床治疗肺癌提供实验依据。

## 1 材料与方法

### 1.1 实验材料

#### 1.1.1 实验细胞和动物

人NSCLC细胞H1299和A549由清华大学江鹏教授提供，小鼠肺癌细胞（lewis lung cells, LLC）购自武汉普诺赛生命科技有限公司。

30只SPF级4周龄雄性C57BL/6 J小鼠，购自宁夏医科大学实验动物中心，实验动物伦理号为：IACU-NYLAC- 2022-029，在宁夏医科大学实验动物中心SPF级别的动物房中进行饲养。

#### 1.1.2 实验药材和试剂

该中药配方的总重量为200 g，由连翘（20 g）、苦参（15 g）、天葵子（15 g）、蔓荆子（20 g）、桂枝（10 g）、虎杖（20 g）、蛇床子（20 g）、厚朴（15 g）、生姜（10 g）、重楼（20 g）、紫草（15 g）、补骨脂（20 g）12味中药组成，均购自宁夏大河源中药有限公司。所有原料均用水煎煮2次，浓缩至400 mL，生药浓度为500 mg/mL，在实验前用0.22 μm PES膜过滤器过滤药物。Dulbecco’s modified eagle’s medium DMEM（C11995500BT）、RPMI-1640（C11875500BT）培养基、预染色蛋白Marker（26616）购自美国Thermo Fisher Scientific；胎牛血清FBS（FCS500）购自吉泰依科赛公司；BCA蛋白定量试剂盒（MPK002）购自上海生工生物工程股份有限公司；MTT（M1020）试剂盒、细胞凋亡检测试剂盒（CA1020）购自北京索莱宝生物科技有限公司；ROS检测试剂盒（S9687）购自美国Selleck；鼠抗β-actin抗体（1:5000, BE0021）、山羊抗兔抗体（1:8000, BE0101）、山羊抗鼠抗体（1:8000, BE0102）购自中国EASYBIO公司；兔抗6-磷酸葡萄糖脱氢酶（6-phosphate glucose dehydrogenase, G6PD）（1:3000, 25413-AP）、兔抗6-磷酸葡萄糖酸脱氢酶（6-phosphogluconate dehydrogenase, 6PGD）（1:1000, 14718-1-AP）、兔抗Bcl-2相关X蛋白（Bcl-2-associated X protein, Bax）（1:1000, 50599-2-Ig）、兔抗B淋巴细胞瘤-2（B-cell lymphoma-2, Bcl-2）（1:1000, 12789-1-AP）购自武汉Proteintech。

#### 1.1.3 实验设备

Waters Acquity I-Class UPLC液相色谱仪串联Waters Xevo G2-S高分辨质谱系统（美国Waters公司）；超纯水系统（美国Life Sciences公司）；台式高速冷冻离心机、超净工作台、液氮罐、CO_2_培养箱（美国Thermo Fisher公司）；流式细胞仪（中国深圳唯工生物）；ECL化学发光系统、蛋白质电泳仪、蛋白转印电泳仪（上海Tanon公司）；多功能酶标仪（中国杭州奥盛公司）；超声破碎仪（中国宁波新芝公司）；移液器（德国Eppendorf公司）；细胞计数仪（美国Bio-Rad公司）。

### 1.2 方法

#### 1.2.1 UHPLC-Q-TOF-MS分析

使用Acquity UHPLC系统和Waters Synapt G2-Si Q-TOF对NCHF-01进行鉴定分析。液相色谱条件为Waters ACQUITY UPLC HSS T3柱（100 mm×2.1 mm, i.d., 1.8 μm），以0.1%甲酸水溶液-乙腈为流动相进行梯度洗脱，柱温35 ^o^C，流速0.25 mL/min，上样量10 μL，检测波长210-400 nm。质谱条件为电喷雾离子源（electrospray ionization, ESI），扫描范围为50-1500 m/z；扫描模式为MSe，温度为500 ^o^C（ESI+）和400 ^o^C（ESI-），-2.5 kV（ESI-）和2.0 kV。

#### 1.2.2 动物模型的建立

将小鼠随机分为6组，每组5只，将1×10^6^ LLC细胞用0.1 mL磷酸盐缓冲液（phosphate-buffered saline, PBS）重悬，皮下注射到小鼠背部一侧。7 d后，对照组给予纯净水灌胃，NCHF-01组分别给予3.75、7.5、15 g/kg药物灌胃，阳性对照组给予5-氟尿嘧啶（5-fluorouracil, 5-FU）25 mg/kg腹腔注射，PBS对照组给予相同剂量腹腔注射，两天一次。15 d后，取出肿瘤并称重。

#### 1.2.3 苏木素-伊红（hematoxylin-eosin, HE）染色

小鼠处理15 d后，乙醚麻醉，0.9%生理盐水心内灌注至肝脏变白，用4%多聚甲醛溶液全身灌注，取出组织置于4%甲醛溶液中过夜。组织包埋于石蜡中，并切成5 µm，置于二甲苯中脱烃，在分级乙醇中再水化，然后用HE染色。使用显微镜拍摄并记录。

#### 1.2.4 细胞培养

H1299细胞采用RPMI-1640培养基，A549和LLC细胞采用DMEM培养基，所有细胞均用含有10%胎牛血清的完全培养基，在37^ o^C、5% CO_2_培养箱中培养。

#### 1.2.5 MTT法检测细胞活性

将5×10^3^个细胞接种到96孔板中，每个处理组6个重复，在37^ o^C、5% CO_2_的培养箱中培养12 h后加入不同浓度药物处理，NCHF-01的浓度为：0、2.5、5.0、7.5、10.0、15.0、20.0、22.5 mg/mL，对照组用水处理，48 h后，用MTT试剂盒检测细胞活力。

#### 1.2.6 结晶紫染色法检测细胞增殖

将2×10^4^个细胞接种在6孔板中，每个处理组4个重复，在37 ^o^C、5% CO_2_的培养箱中培养。细胞贴壁后，加入不同浓度NCHF-01处理，每隔1天更换含有NCHF-01的培养基，对照组用水处理。7 d后，选取每个处理组中的3个重复，使用细胞计数仪统计细胞数，1个重复用4%多聚甲醛固定细胞15 min，用0.05%结晶紫染色15 min，PBS清洗后拍照。

#### 1.2.7 细胞周期检测

NCHF-01处理细胞48 h后，收集细胞，70%乙醇，4^ o^C固定12 h后，加入100 μL RNase A在37 ^o^C下孵育30 min，加入400 μL PI，在4^ o^C下避光孵育30 min。使用流式细胞仪检测细胞周期，利用Flow Jo对数据进行分析。

#### 1.2.8 细胞凋亡检测

NCHF-01处理细胞48 h后，收集细胞，加入100 μL的1×Binding Buffer重悬细胞，再加入5 μL的AV试剂避光染色10 min，最后加入5 μL的PI试剂避光染色5 min，补加900 μL的1×Binding Buffer。使用流式细胞仪检测细胞凋亡，利用Flow Jo对数据进行分析。

#### 1.2.9 网络药理学分析

将UHPLC-Q-TOF-MS分析得到的化合物使用Swiss Target Prediction预测其治疗靶点。通过GeneCards和DisGeNET数据库收集NSCLC相关的疾病靶点。利用Venny 2.1.0软件筛选化合物靶点与疾病靶点的交集。利用STRING数据库构建蛋白与蛋白互作（protein-protein interaction, PPI）网络。采用基因本体（Gene Ontology, GO）和京都基因与基因组百科全书（Kyoto Encyclopedia of Genes and Genomes, KEGG）富集分析对化合物作用于NSCLC的分子机制进行预测。

#### 1.2.10 活性氧测定

NCHF-01处理细胞H1299和A549，48 h后，加入含有10 µmol/L DCFH-DA的无血清培养基，在37^ o^C、5% CO_2_培养箱中孵育30 min。收集细胞，PBS重悬，使用流式细胞仪检测细胞内活性氧（reactive oxygen species, ROS）水平，利用Flow Jo对数据进行分析。

#### 1.2.11 Western blot检测相关蛋白表达

NCHF-01处理细胞H1299和A549，48 h后，提取细胞总蛋白，BCA测定蛋白浓度。使用相同浓度蛋白上样量进行凝胶电泳，转移到硝化纤维素膜上，5%脱脂牛奶封闭1 h，一抗4 ^o^C孵育过夜，使用TBST清洗3遍，每遍4 min，室温孵育二抗1 h，TBST清洗3遍，每遍4 min，ECL发光液孵育，化学发光仪曝光显影。

#### 1.2.12 G6PD和6PGD酶活性检测

NCHF-01处理细胞H1299和A549，48 h后，加入100 μL的EMBOIP Buffer裂解液，冰上裂解30 min，离心去沉淀，BCA测定蛋白浓度，并将蛋白量调整为一致，取20 μL加入到180 μL的缓冲体系（1 mmol/L MgCl_2_，50 mmol/L Tris pH 8.1，200 µmol/L G6PD和6PGD，100 μmol/L NADP^+^）中，利用酶标仪，在341 nm下进行酶活性检测，每15 s检测1次，检测14 min。测定结果为还原型烟酰胺腺嘌呤二核苷酸磷酸（nicotinamide adenine dinucleotide phosphate, NADPH）的含量，以此测定G6PD和6PGD的酶活性。

#### 1.2.13 免疫组织化学（immunohistochemistry, IHC）

采用IHC检测Ki-67、Bcl-2、G6PD、6PGD在肿瘤组织中的表达。按照说明书进行，脱蜡和再复水石蜡切片与Ki-67（1:500）、Bcl-2（1:300）、G6PD（1:300）、6PGD（1:300）一抗孵育2 h后，与相应的二抗孵育2 h，然后，滴加新鲜的二甲基联苯胺底物对切片进行可视化，使用苏木素反染，并进行镜检分析。

### 1.3 统计学分析

采用GraphPad Prism软件进行分析并作图，数据均以均数±标准差（Mean±SD）表示。两组之间差异比较采用t检验。以P<0.05为差异有统计学意义。

## 2 结果

### 2.1 NCHF-01中活性成分分析

NCHF-01的UPLC-Q-TOF/MS的代表性色谱图见图1。经过分析，利用数据库匹配和现有文献对NCHF-01的109个活性成分进行初步鉴定和描述。从NCHF-01中鉴定出的主要活性成分，包括37种黄酮类化合物、13种皂苷类化合物、8种香豆素类化合物、7种羧酸类化合物、8种生物碱类化合物、6种苷类化合物、14种酚类化合物、种木质素类化合物、7种醌类化合物以及其他类型化合物。其中虎杖、连翘和重楼是鉴定活性成分的主要来源。之后，我们选取NCHF-01中几种活性成分进行定量分析（表1），结果显示，在NCHF-01中和厚朴酚的平均浓度为20.9 ng/mL，氧化苦参碱的平均浓度为39.5 ng/mL，重楼皂苷VII、连翘酯苷A及虎杖甙的平均浓度分别达到249.7、354.7以及381.7 ng/mL。

**图1:**
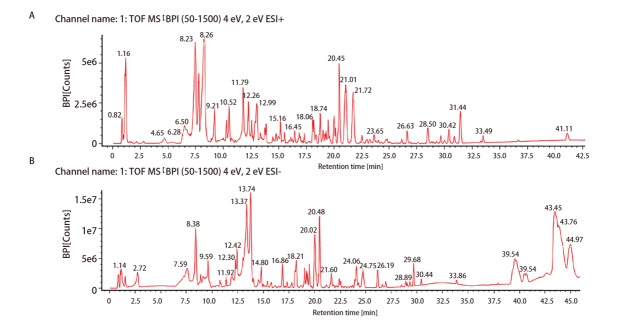
NCHF-01的UPLC-Q-TOF-MS基峰离子流图。A：正离子模式；B：负离子模式。

**表1:** NCHF-01成分分析结果

Lot	Component	Molecular formula	Linear equation	R	Retention time (min)	Concentration (ng/mL)
20230201	Honokiol	C_18_H_18_O_2_	Y=2.19e+004x-497	0.9979	7.18	41.80
	Forsythiaside A	C_29_H_36_O_15_	Y=1.06e+004x-266	0.9980	3.43	7.10×10^5^
	ChongLou Saponin VII	C_51_H_82_O_21_	Y=316.00x+5.04	0.9976	7.31	508.00
	Polydatin	C_20_H_22_O_8_	Y=3.47e+004x+290	0.9995	3.32	1.90×10^5^
	Oxymatrine	C_15_H_24_N_2_O_2_	Y=3.93e+004x+4.94e+003	0.9980	1.83	7.64×10^4^
20230413	Honokiol	C_18_H_18_O_2_	Y=2.19e+004x-497	0.9979	7.18	43.00
	Forsythiaside A	C_29_H_36_O_15_	Y=1.06e+004x-266	0.9980	3.43	7.04×10^5^
	ChongLou Saponin VII	C_51_H_82_O_21_	Y=316.00x+5.04	0.9976	7.31	498.00
	Polydatin	C_20_H_22_O_8_	Y=3.47e+004x+290	0.9995	3.32	1.90×10^5^
	Oxymatrine	C_15_H_24_N_2_O_2_	Y=3.93e+004x+4.94e+003	0.9980	1.83	7.62×10^4^
20230703	Honokiol	C_18_H_18_O_2_	Y=2.19e+004x-497	0.9979	7.18	40.60
	Forsythiaside A	C_29_H_36_O_15_	Y=1.06e+004x-266	0.9980	3.43	7.14×10^5^
	ChongLou Saponin VII	C_51_H_82_O_21_	Y=316.00x+5.04	0.9976	7.31	492.00
	Polydatin	C_20_H_22_O_8_	Y=3.47e+004x+290	0.9995	3.32	1.94×10^5^
	Oxymatrine	C_15_H_24_N_2_O_2_	Y=3.93e+004x+4.94e+003	0.9980	1.83	7.54×10^4^

### 2.2 NCHF-01抑制LLC荷瘤小鼠肿瘤的生长

为了评估NCHF-01是否能够达到治疗NSCLC的效果，我们构建LLC荷瘤小鼠模型，并通过灌胃的方式进行给药处理，15 d后取出肿瘤并拍照。研究结果表明，与对照组相比，NCHF-01明显抑制了LLC荷瘤小鼠的肿瘤生长（图2A，图2B）。为检测NCHF-01是否存在副作用，我们将LLC荷瘤小鼠心脏、肝脏和肾脏取出，并进行HE染色。结果表明，在NCHF-01治疗组中，没有发现明显的组织学改变（图2C）。此外，我们还检测了血液中丙氨酸氨基转移酶、天门冬氨酸氨基转移酶、血尿素氮、肌酐和碱性磷酸酶的含量，以了解NCHF-01是否影响肝脏和肾脏功能。结果显示，在NCHF-01治疗组和未治疗组之间没有发现明显的差异（表2）。以上结果显示，该中药配方NCHF-01能够抑制肿瘤的生长，并且无明显的毒副作用。

**图2:**
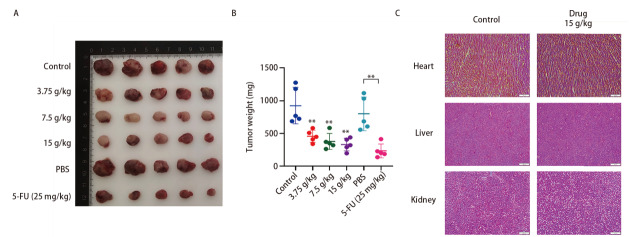
NCHF-01对LLC荷瘤小鼠肿瘤生长的影响。A、B: 不同处理组小鼠的肿瘤大小（A）及重量（B）；C: 小鼠不同器官组织切片HE染色（比例尺=200 µm）。**P<0.01。

**表2:** NCHF-01对肿瘤小鼠肝肾功能的影响

Item	Control	NCHF-01 (15 g/kg）	P
ALT (U/L)	46.60±2.61	47.60±3.29	0.6085
AST (U/L)	133.67±10.41	131.00±20.66	0.9262
BUN (mmol/L)	7.41±0.75	6.89±0.48	0.8263
CRE (mmol/L)	6.75±1.26	6.25±0.50	0.4881
ALP (U/L)	139.00±19.77	135.75±10.22	0.8267

ALT: alanine aminotransferase; AST: aspartate aminotransferase; BUN: blood urea nitrogen; CRE: serum creatinine; ALP: alkaline phosphatase.

### 2.3 NCHF-01抑制NSCLC细胞的增殖

通过构建小鼠肿瘤模型，我们测定该中药配方NCHF-01能够抑制肿瘤的生长（图2）。因此，我们通过细胞实验进一步验证该中药配方所发挥的抗肿瘤作用。为了检测NCHF-01对NSCLC细胞活力的影响，我们利用MTT法检测细胞活力，结果显示，在药物的作用下，H1299和A549细胞的细胞活力明显下降，NCHF-01能够明显抑制细胞的活力（图3A，图3B），其对H1299和A549细胞的半数抑制浓度（half maximal inhibitory concentration, IC_50_）分别为8.9和9.6 mg/mL，由于10.0和15.0 mg/mL之间没有统计学意义上的差异，我们选择5.0、10.0 mg/mL进行后续实验。为了验证NCHF-01对NSCLC细胞的抗增殖作用，我们进行结晶紫染色实验，结果显示，对照组（0 mg/mL）中H1299和A549细胞数分别为8.29×10^5^和9.12×10^5^，在使用不同浓度药物处理后，5.0 mg/mL中H1299和A549细胞数分别为3.31×10^5^和4.02×10^5^，10.0 mg/mL中H1299和A549细胞数分别为8.36×10^4^和7.88×10^4^，细胞的增殖能力明显下降（图3C，图3D）。表明NCHF-01能够抑制NSCLC细胞的增殖能力。

**图3:**
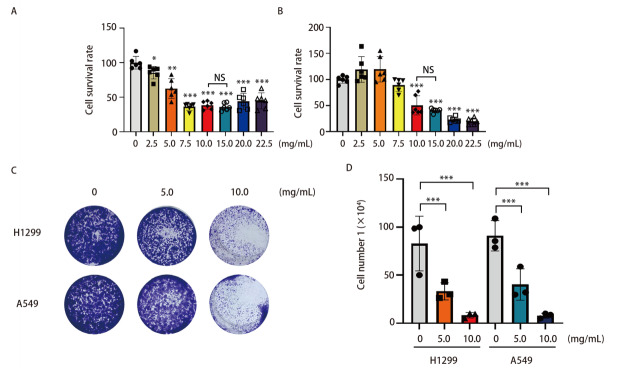
NCHF-01对NSCLC细胞增殖的影响。A、B：NCHF-01以0、2.5、5.0、7.5、10.0、15.0、20.0、22.5 mg/mL浓度处理细胞48 h后，检测H1299（A）和A549（B）细胞的活力；C、D: NCHF-01以0、5.0、10.0 mg/mL浓度处理细胞，7 d后观察其对细胞增殖的影响（C）并计数（D）。*P<0.05；**P<0.01；***P<0.001。

### 2.4 NCHF-01抑制NSCLC细胞周期，并促进细胞凋亡

为了进一步探讨NCHF-01对细胞增殖的抑制是否是由于其对细胞周期的影响，我们进行流式细胞术实验去检测细胞周期的变化，结果显示，NCHF-01促进细胞的G_2_/M期停滞（图4A，图4B）。之后，我们利用Annexin V-FITC和Propidium iodide对细胞进行双重标记，以检测NCHF-01对细胞凋亡的影响，结果显示，与对照组相比，H1299和A549细胞的凋亡率随药物浓度的增加呈现上升趋势，从11.01%、9.79%上升至64.90%、62.90%，其明显增加了H1299和A549细胞的凋亡率（图4C，图4D）。结果表明，NCHF-01处理可以有效地诱导NSCLC细胞的G_2_/M期停滞，抑制细胞周期，并促进细胞凋亡。

**图4:**
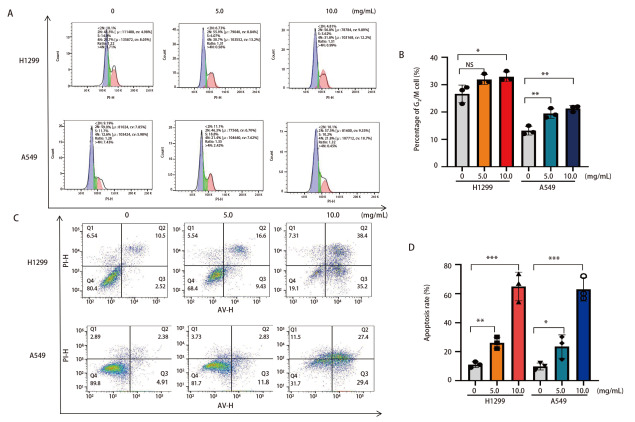
NCHF-01以0、5.0、10.0 mg/mL浓度处理细胞48 h后，流式细胞术检测H1299和A549细胞的细胞周期（A、B）和细胞凋亡（C、D）。*P<0.05；**P<0.01；***P<0.001。

### 2.5 网络药理学预测NCHF-01治疗NSCLC的作用机制

为了探究该中药配方抑制NSCLC的分子机制，我们利用网络药理学预测NCHF-01治疗NSCLC的作用靶点。从GeneCards数据库和DisGeNET数据库中分别收集得到2655个和13个候选靶点，删除重复后得到2657个NSCLC相关基因靶点。将UHPLC-Q-TOF-MS分析得到的NCHF-01中含有的活性成分使用Swiss Target Prediction预测，得到1034个治疗所有疾病的靶点。利用Venny 2.1对2657个NSCLC相关疾病靶点基因和1034个预测的NCHF-01治疗所有疾病的靶点进行映射和交叉，得到464个NCHF-01治疗NSCLC的靶点（图5A）。根据选定的活性成分和药物靶点，构建“成分-疾病-靶点”网络，将464个相关靶点导入STRING数据库，获得其相互作用关系，通过Cytoscape软件进行可视化，获得PPI网络（图5B）。GO分析（图5C）显示NCHF-01通过各种生物学过程影响NSCLC的发生和发展，包括肽-酪氨酸磷酸化、激酶活性的正向调节、丝裂原活化蛋白激酶（mitogen activated protein kinase, MAPK）级联的正向调节、氧化应激反应、细菌来源分子的反应、蛋白激酶活性的正调控、腺体发育、活性氧反应、细胞因子产生的正向调节。KEGG分析显示（图5D），磷脂酰肌醇3-激酶/丝氨酸-苏氨酸蛋白激酶B（phosphatidylinositol 3-kinase/protein kinase B, PI3K/AKT）信号通路、细胞凋亡、MAPK信号通路、脂质和动脉粥样硬化、内分泌抵抗、碳代谢等是参与NCHF-01治疗NSCLC的主要代谢通路。网络药理学结果显示，氧化应激以及碳代谢在NCHF-01的抗NSCLC中发挥重要作用。

**图5:**
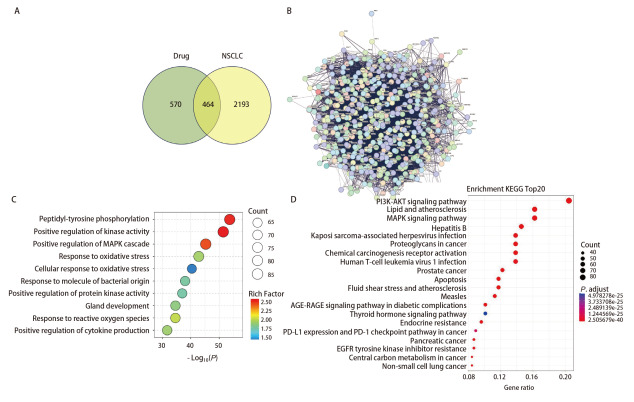
NCHF-01的网络药理学分析结果。A：Venny图交集得到的NCHF-01治疗NSCLC的靶点；B：根据交集得出的作用靶点构建PPI网络；C、D：交集靶点的GO分析与KEGG富集分析。

### 2.6 NCHF-01诱导NSCLC细胞ROS的积累

为了维持稳定的肿瘤微环境，肿瘤细胞需调节各种抗氧化机制。研究表明，ROS水平异常高时，它可以通过与线粒体蛋白的相互作用引发肿瘤细胞的凋亡。因此，我们用5.0和10.0 mg/mL浓度的NCHF-01处理H1299和A549细胞，并检测细胞内ROS水平。结果显示，与对照组相比，NCHF-01处理组的ROS水平随药物浓度的增加也呈现上升趋势，明显增加了细胞内ROS的水平（图6A，图6B）。为了研究ROS在NCHF-01的抗NSCLC中的作用，我们用ROS清除剂N-乙酰半胱氨酸（N-acetylcysteine, NAC）处理H1299和A549细胞，与对照组相比，加入NAC后，细胞内ROS水平明显下降，并且细胞凋亡率出现明显下降，降低了NCHF-01诱导的ROS积累（图6C，图6D）和细胞凋亡（图6E，图6F）。结果表明，ROS的积累能够促进NCHF-01的抗NSCLC作用。

**图6:**
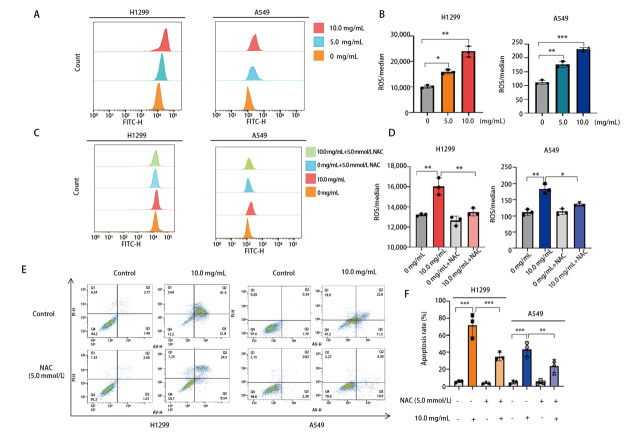
NCHF-01对NSCLC细胞中ROS水平的影响。A、B：NCHF-01以0、5.0、10.0 mg/mL浓度处理细胞48 h后，检测细胞ROS水平的变化；C、D：NCHF-01以0、10.0 以及5.0 mmol/L NAC浓度处理细胞48 h后，检测细胞ROS水平的变化；E、F：NCHF-01以0、10.0以及5.0 mmol/L NAC浓度处理细胞48 h后，检测细胞凋亡的变化。*P<0.05；**P<0.01；***P<0.001。

### 2.7 NCHF-01抑制NSCLC细胞的磷酸戊糖途径（pentose phosphate pathway, PPP）

根据网络药理学分析得出，氧化应激以及碳代谢在NCHF-01的抗NSCLC中发挥重要作用，而在碳代谢过程中，PPP所产生的NADPH对调节细胞氧化还原平衡至关重要，我们假设NCHF-01通过抑制NSCLC细胞中的PPP，进而降低NADPH的水平，导致细胞内ROS的积累。因此，我们用NCHF-01处理H1299和A549细胞，并检测PPP关键酶的表达。结果表明，与对照组相比，NCHF-01降低了PPP中关键酶G6PD和6PGD的蛋白表达和抑凋亡蛋白Bcl-2的表达，促进了促凋亡蛋白Bax的表达（图7A），并减弱细胞中G6PD和6PGD的酶活性（图7B）。此外，我们提取LLC荷瘤小鼠模型肿瘤组织蛋白，检测肿瘤组织中PPP和凋亡相关蛋白的表达水平，进一步研究NCHF-01的抗肿瘤作用。结果显示，与对照组小鼠相比，NCHF-01治疗组的G6PD、6PGD和Bcl-2的蛋白表达较低，Bax的蛋白表达则较高（图7C）。通过IHC分析检测Ki-67、G6PD和6PGD的表达，如图7D所示，NCHF-01治疗明显降低了Ki-67和Bcl-2的表达，表明NCHF-01可以抑制NSCLC细胞的生长，并且G6PD和6PGD的表达减少表明NCHF-01诱导了PPP的抑制。以上结果表明，NCHF-01可以抑制NSCLC细胞的PPP，导致细胞内氧化还原失衡，引起ROS的积累，进而促进细胞凋亡。

**图7:**
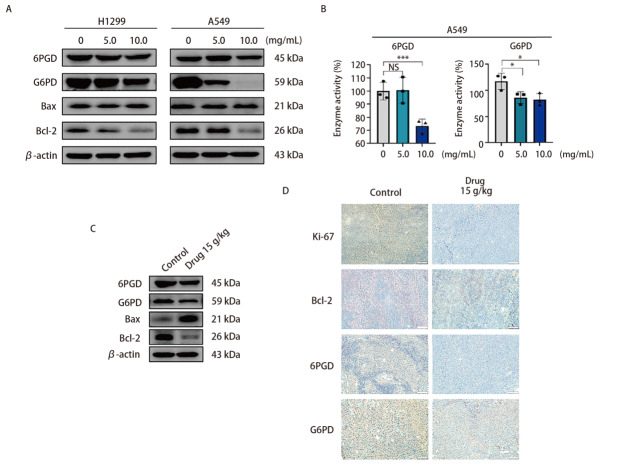
NCHF-01对NSCLC的PPP的影响。A、B：NCHF-01以0、5.0、10.0 mg/mL浓度处理细胞48 h后，Western blot检测PPP相关蛋白表达（A），并检测PPP关键酶活性（B）；C：Western blot检测不同处理组小鼠肿瘤组织PPP相关蛋白表达；D：IHC检测肿瘤组织Ki-67、Bcl-2、G6PD和6PGD的表达（比例尺=200 µm）。*P<0.05；***P<0.001。

## 3 讨论

根据临床发现，NCHF-01具有抗NSCLC的作用，能够抑制肺部肿瘤的生长，并帮助血液指数恢复正常。然而，其治疗肺癌的作用机制仍然是未知的。通过体内外实验，我们验证该中药配方NCHF-01具有抗NSCLC能力。网络药理学分析得知，氧化应激以及碳代谢在NCHF-01的抗NSCLC中可能发挥重要作用。

一般来说，适量的ROS会激活许多信号通路，导致正常细胞发生增殖和癌变。细胞内过量的ROS通过对细胞大分子如蛋白质、脂质和DNA等造成损伤而导致细胞死亡^[11⇓⇓-14]^。ROS可以减少促凋亡因子Bax的泛素化，增加抗凋亡因子Bcl-2的泛素化，降低Bcl-2与Bax的比例^[15⇓-17]^。因此，增加ROS的积累以特异性杀伤癌细胞和清除异常升高的ROS以防止癌症形成都是有益的抗癌策略。网络药理学结果表明，NCHF-01治疗NSCLC与氧化应激和中枢碳代谢有关。通过细胞实验证明，NCHF-01能够促进细胞中ROS水平的升高。接下来，我们发现使用5 mmol/L ROS清除剂NAC处理可以减少NCHF-01诱导的ROS增加并抑制NSCLC细胞凋亡，表明ROS可能有助于NCHF-01诱导的NSCLC细胞凋亡。

当产生ROS的过程和减少ROS的过程不平衡时，ROS水平会增加。低水平的ROS实际上促进了肿瘤细胞的增殖、生长、侵袭和转移^[18,19]^。然而，异常增加的ROS可以与线粒体蛋白相互作用，诱导肿瘤细胞凋亡^[18]^。NADPH是一种主要的细胞内氢供体，对清除ROS至关重要。NADPH能够参与细胞合成代谢反应和氧化还原平衡，癌细胞增加其NADPH水平免受ROS损害^[20,21]^，而细胞质中的NADPH主要由G6PD和6PGD通过PPP途径产生^[22]^。肿瘤细胞的代谢被重新编程，以实现强大的抗氧化防御和生物合成^[23,24]^，使PPP成为满足细胞抗氧化防御和生物合成需求的关键葡萄糖代谢途径^[25]^。G6PD是PPP第一步中的关键酶，为细胞提供NADPH，用于还原性抗氧化防御和生物合成。我们的实验结果显示NCHF-01降低G6PD和6PGD的蛋白表达和酶活性。因此，NCHF-01对PPP的抑制减少了细胞对葡萄糖的摄取和NADPH的生成，导致细胞内ROS的积累，有助于NCHF-01促进NSCLC细胞的凋亡。

我们的研究表明，NCHF-01可能是一种ROS诱导剂，进而促进NSCLC细胞发生凋亡。大规模、多中心合作的临床试验在未来对优化NCHF-01在临床上的应用潜力至关重要。由于时间限制，尚未分析NCHF-01的哪种活性成分会影响NSCLC的PPP调节。然而，通过对这些物质的附加和协同作用的额外研究，这一结果将得到改善。在这项研究中，结果显示，NCHF-01对NSCLC细胞和LLC荷瘤小鼠具有明显的治疗效果。通过网络药理学，我们发现NCHF-01的药理作用受到ROS水平和PPP途径的影响。因此，我们的研究为进一步利用NCHF-01治疗NSCLC提供了一个坚实的理论基础。

伦理声明

本研究得到了动物保护机构和使用委员会的批准（Certificate No.IACU-NYLAC-2022-029）。

Competing interests

The authors declare that they have no competing interests.

Author contributions

Cheng C and Wu W performed the experiments, analyzed the data and wrote the draft manuscript. Yu JX and Yuan DD carried out data curation. Li L and Wang YJ provided critical inputs on design, analysis, and interpretation of the study. All the authors had access to the data. All authors read and approved the final manuscript as submitted.
